# Genotype Frequencies of 50 Polymorphisms for 241 Japanese Non-cancer Patients

**DOI:** 10.2188/jea.12.229

**Published:** 2007-11-30

**Authors:** Nobuyuki Hamajima, Toshiko Saito, Keitaro Matsuo, Takashi Suzuki, Tsuneya Nakamura, Akio Matsuura, Katashi Okuma, Kazuo Tajima

**Affiliations:** 1 Division of Epidemiology and Prevention, Aichi Cancer Center Research Institute.; 2 Nagoya University Graduate School of Medicine.; 3 Department of Gastroenterology, Aichi Cancer Center Hospital.; 4 Department of Clinical Laboratory, Aichi Cancer Center Hospital.

**Keywords:** polymorphisms, genotype frequencies, PCR-CTPP

## Abstract

This paper lists the genotype frequencies of 50 polymorphisms of 37 genes (*ALDH2*, *ADRB2*, *ADRB3*, *COMT*, *CD36*, *CXCR2*, *CCND1*, *COX2*, *CYP2A6*, *CYP17*, *CYP19*, *IGF1*, *IL-1A*, *IL-1B*, *IL-1RN*, *IL-1R1*, *IL-6*, *IL-8*, *IL-10*, *LEP*, *Le*, *L-myc*, *MPO*, *MTR*, *MTHFR*, *MAO-A*, *NQO1*, *OGG1*, *p53*, *p73*, *Se*, *SRD5A2*, *TGF-B*, *TNF-A*, *TNF-B*, *XPD*, and *XRCC1*) and 6 sets of combined genotype frequencies for 241 non-cancer Japanese outpatients. Though the genotype frequencies of 25 polymorphisms have already been reported in our previous papers, 15 polymorphisms (*CD36* *A52C*, *CXCR2* C785T, *CCND1 G870A*, *IGF1* C/T at intron 2 and G2502T, *IL-1A* 46-bp VNTR, *IL-1R1* C-116T, *IL-6* Ins/Del 17C, *IL-8* A-278T and C74T, *IL-10* T-819C, *LEP* A-2548G, *SRD5A2* 2-bp VNTR, *XPD* Lys751Gln, and *XRCC1* Arg399Gln) and six sets of combined genotype frequencies (*IL-1B* C-31T and *IL-1A* C-889T, *IL-1B* C-31T and *IL-1RN* 86-bp VNTR, *IL-1B* C-31T and *IL-1R1* C-116T, *TNF-A* G-308A and *TNF-B* A252G, *SRD5A2* Val89Leu and 2-bp VNTR, and *XRCC1* Arg399Gln and *XPD* Lys751Gln) were reported in this paper for the first time for Japanese. Although microarray technology will produce this kind of information in near future, this is the first document that reports the genotype/allele frequencies among Japanese for an archival purpose.

As polymerase chain reaction (PCR) techniques develop, polymorphism studies to measure associations with disease risk have been rapidly increasing. The epidemiologic purposes are to identify high risk individuals and further to detect interactions between genetic traits and environmental exposure for disease risk. The polymorphism studies also provide clues to elucidate biological mechanisms of diseases, because the observed associations indicate that the genes may play a pivotal role for disease occurrence.

Through the studies for different ethnic groups, it has found that allele frequencies of some polymorphisms vary group by group. For example, *A* allele of *tumor necrosis factor A* (*TNF-A*) at -308 was reported to be 1.7% for Japanese (n=575)^1^^)^ and 16.5% for Caucasians (n=106)^2^^)^, suggesting that *TNF-A* A-308G studies for Japanese require a larger sample size and that the social impact is smaller than that for Caucasians. Therefore, allele frequencies for each area or ethnic group are useful information when polymorphism studies are designed.

This paper reports genotype and allele frequencies of the 50 polymorphisms genotyped for 241 non-cancer patients of Aichi Cancer Center Hospital. In addition, 6 sets of combined genotype frequencies of polymorphisms making a cluster are presented. Although genotype frequencies for 25 polymorphisms have been reported in our previous papers^3^^-^^14^^)^, they were also included because of the importance in the archival role of this report. Though a database of single nucleotide polymorphisms (SNPs) for Japanese is accessible at the home page of JSNP (http://snp.ims.u-tokyo.ac.jp/index_ja.html), the data are based on only 24 individuals, and there are no data on the linkage between SNPs. National Center for Biotechnology Information (NCBI) in the United States also provide a database of SNPs registered mainly by National Human Genome Research Institute (http://www.ncbi.nlm.nih.gov/SNP/), but not necessarily for Japanese.

## SUBJECTS AND METHODS

Subjects were 241 non-cancer outpatients of Aichi Cancer Center Hospital, who participated in *Helicobacter pylori* eradication study enrolled between March and December in 1999. They included 97 (40.2% out of 241) participants who stated to be under medication for 107 diseases (not confirmed by their medical records); 23 with gastric/duodenal ulcer, another 23 for so-called gastritis, 16 with hypertension, 8 for pain including arthritis and lumbago, 7 with diabetes mellitus, 7 with hyperlipidemia, 3 with an ischmic heart disease, 3 with a thyroid disease, 2 with a gynecological disease, 2 with hyperuricaemia, 2 with Meniere disease, 2 with a prostate disease, 1 with ulcerative colitis, 1 with pancreatitis, 1 with asthma, 1 with arrhythmia, 1 for epilepsy, 1 for neurosis, 1 with liver cirrhosis, 1 for ulticaria, and 1 after cerebral infarction^15^^)^. All the participants provided written informed consent before they donated a 7ml blood sample from peripheral vein.

Selected were 50 polymorphisms successfully genotyped until September 2001, for the purpose of case-control studies by our research group. All were polymorphisms discovered by other researchers. A SNP registration number is attached for unpublished polymorphisms. The abbreviations of the genes are listed on Table 1. The alleles of variable number of tandem repeat (VNTR) polymorphisms were described by the number of repeats. For example, the allele of 86-base pair (bp) VNTR polymorphism of *IL-1RN* is described 2, 3, 4, or 5 (repeats). We have been conducted case-control studies for malignant lymphoma and cancers of the esophagus, stomach, colorectum, lung, and breast, as well as *Helicobacter pylori* infection and smoking habit. The order of polymorphism selection was based on the priority of the studies, as well as easiness for genotyping. All the polymorphisms were those which produce DNA bands with a different length distinguishable by agarose gel electrophoresis.

**Table 1.** Genotype and allele frequencies of 50 genetic polymorphisms for 241 non-cancer patients.

No.	Gene	Location (Accession No.)	Polymorphism	Genotyping	N	Frequency (%)						
						Genotype				Allele	
1′	*Aldehydodehydrogenase 2* (*ALDH2*)	12q24	Glu487Lys	PCR-CTPP	241	*Glu/Glu*	*Glu/Lys*	*Lys/Lys*		*Glu*	*Lys*	
		(NT_000613)				52.3	39.8	7.9		72.2	27.8	
2′	*Beta-adrenoceptor 2* (*BAR2*, *ADRB2*)	5q31-32	Gln27Glu	PCR-CTPP	239	*Gln/Gln*	*Gln/Glu*	*Glu/Glu*		*Gln*	*Glu*	
		(NM_000024)				83.3	16.3	0.4		91.4	8.6	
3′	*Beta-adrenoceptor 3 *(*BAR3*, *ADRB3*)	8p11-12	Trp64Arg	PCR-RFLP	239	*Trp/Trp*	*Trp/Arg*	*Arg/Arg*		*Trp*	*Arg*	
						64.4	32.2	3.3		80.5	19.5	
4′	*Catechol-O-methyltransferase* (*COMT*)	22q11.2	Val158Met	PCR-RFLP	123^F^	*Val/Val*	*Val/Met*	*Met/Met*		*Val*	*Met*	
						46.3	38.2	15.4	*	65.4	34.6	
5	*CD36*	7q11.2	Pro90Ser	PCR-CTPP	240	*Pro/Pro*	*Pro/Ser*	*Ser/Ser*		*Pro*	*Ser*	
		(L06849)				91.7	8.3	0.0		95.8	4.2	
6			A52C	PCR-CTPP	240	*A/A*	*A/C*	*C/C*		*A*	*C*	
			(JST005702)			52.1	42.5	5.4		73.3	26.7	
7	*CXC chemokine receptor 2* (*CXCR2*)	2q34-35	C785T	PCR-CTPP	235	*C/C*	*C/T*	*T/T*		*C*	*T*	
		(M99412)				46.8	42.6	10.6		68.1	31.9	
8	*Cyclin D1* (*CCND1*)	11q13	G870A	PCR-RFLP	241	*G/G*	*G/A*	*A/A*		*G*	*A*	
						27.4	46.1	26.6		50.4	49.6	
9′	*Cyclooxigenase 2* (*COX2*)	1q25-31	G-765C	PCR-CTPP	241	*G/G*	*G/C*	*C/C*		*G*	*C*	
		(NT_001817)	(dbSNP:20417)			95.4	4.6	0.0		97.7	2.3	
10′			C-163G	PCR-CTPP	237	*C/C*	*C/G*	*G/G*		*C*	*G*	
			(dbSNP:5270)			97.0	3.0	0.0		98.5	1.5	
11′			C-62G	PCR-CTPP	50	*C/C*	*C/G*	*G/G*		*C*	*G*	
			(dbSNP:20424)			100.0	0.0	0.0		100.0	0.0	
12′			T10G	PCR-CTPP	237	*T/T*	*T/G*	*G/G*		*T*	*G*	
			(dbSNP:20425)			97.0	3.0	0.0		98.5	1.5	
13′			Glu488Gly	PCR-CTPP	50	*Glu/Glu*	*Glu/Gly*	*Gly/Gly*		*Glu*	*Gly*	
			(dbSNP:5272)			100.0	0.0	0.0		100.0	0.0	
14′			Val511Ala	PCR-CTPP	50	*Val/Val*	*Val/Ala*	*Ala/Ala*		*Val*	*Ala*	
			(dbSNP:5273)			100.0	0.0	0.0		100.0	0.0	
15	*Cytochorome p450 2A6* (*CYP2A6*)	19q13.2	Wt/Del/Conv	PCR-RFLP	239	*W/W*	*W/C*	*W/D*		*W*	*C*	
						11.7	33.9	15.9		36.6	44.8	
						*C/C*	*C/D*	*D/D*		*D*		
						20.5	14.6	3.3		18.6		
16′	*Cytochrome p450 17* (*CYP17*)	10q24.3	T-34C	PCR-RFLP	123^F^	*T/T*	*T/C*	*C/C*		*T*	*C*	
						27.6	57.7	14.6	*	56.5	43.5	
17	*Cytochrome p450 19* (*CYP19*)	15q21.1	Trp39Arg	PCR-CTPP	241	*Trp/Trp*	*Trp/Arg*	*Arg/Arg*		*Trp*	*Arg*	
		(M30796)				90.5	9.1	0.4		95.0	5.0	
18	*Insulin-like growth factor 1*(*IGF1*)	12q22-24	C/T at intron 2	PCR-CTPP	60	*C/C*	*C/T*	*T/T*		*C*	*T*	
		(NT_002970)	(dbSNP:118397)			100.0	0.0	0.0		100.0	0.0	
19			G2502T 3′UTS	PCR-CTPP	240	*T/T*	*T/G*	*G/G*		*T*	*G*	
			(dbSNP:6217)			54.6	37.5	7.9		73.3	26.7	
20′	*Interleukin 1A* (*IL-1A*)	2q14	C-889T	PCR-CTPP	241	*C/C*	*C/T*	*T/T*		*C*	*T*	
		(X03833)				83.4	16.2	0.4		91.5	8.5	
21			46-bp VNTR	PCR	241	*4*	*5*			*4*	*5*	
						100.0	0.0			100.0	0.0	
22′	*Interleukin 1B* (*IL-1B*)	2q14	C-31T	PCR-CTPP	241	*C/C*	*C/T*	*T/T*		*C*	*T*	
		(X04500)				17.4	55.2	27.4	*	45.0	55.0	
23′			C-511T	PCR-RFLP	239	*C/C*	*C/T*	*T/T*		*C*	*T*	
						28.9	54.0	17.2		55.9	44.1	
24′	*Interleukin 1RN* (*IL-1RN*)	2q14.2	86-bp VNTR	PCR	241	*2/2*	*2/4*	*2/5*		*2*	*3*	
						0.4	7.1	0.4		4.1	0.2	
						*3/4*	*4/4*	*4/5*		*4*	*5*	
						0.4	90.0	1.7		94.6	1.0	
25	*Interleukin 1 receptor 1* (*IL-1R1*)	2q12-13	C-116T	PCR-CTPP	239	*C/C*	*C/T*	*T/T*		*C*	*T*	
		(U14179)				38.9	47.7	13.3		62.8	37.2	
26	*Interleukin 6* (*IL-6*)	7p21	Ins/Del 17C	PCR-CTPP	57	*Ins/Ins*	*Ins/Del*	*Del/Del*		*Ins*	*Del*	
		(AF039337)				100.0	0.0	0.0		100.0	0.0	
27	*Interleukin 8* (*IL-8*)	4q12-13	T-278A	PCR-CTPP	235	*T/T*	*T/A*	*A/A*		*T*	*A*	
		(AF385628)				50.2	40.4	9.4		70.4	29.6	
28			C74T	PCR-CTPP	100	*C/C*	*C/T*	*T/T*		*C*	*T*	
						100.0	0.0	0.0		100.0	0.0	
29	*Interleukin 10* (*IL-10*)	1q31-32	T-819C	PCR-CTPP	241	*T/T*	*T/C*	*C/C*		*T*	*C*	
		(U16720)				45.6	44.8	9.5		68.0	32.0	
30	*Leptin* (*LEP*)	7q31.3	A-2548G	PCR-CTPP	237	*A/A*	*A/G*	*G/G*		*A*	*G*	
		(U43589)				60.8	36.3	3.0		78.9	21.1	
31′	*Lewis gene* (*Le*, *FUT3*)	19p13.3	Le(Le,le3)/le(le1,le2)	PCR-RFLP	239	*Le/Le*	*Le/le*	*le/le*		*Le*	*le*	
						51.9	41.0	7.1		72.4	27.6	
32′	*L-myc*	1p34.3	L/S	PCR-RFLP	241	*L/L*	*L/S*	*S/S*		*L*	*S*	
						24.5	55.6	19.9	*	52.3	47.7	
33′	*Myeloperoxidase* (*MPO*)	17q21-23	G-463A	PCR-RFLP	241	*G/G*	*G/A*	*A/A*		*G*	*A*	
						79.7	19.5	0.8		89.4	10.6	
34′	*Methionine synthase* (*MTR*)	1q43	A2756G:Asp/Gly	PCR-RFLP	241	*A/A*	*A/G*	*G/G*		*A*	*G*	
						64.7	32.8	2.5		81.1	18.9	
35′	*Methylenetetrahydrofolate reductase* (*MTHFR*)	1p36.3	C677T:Ala223Val	PCR-RFLP	241	*C/C*	*C/T*	*T/T*		*C*	*T*	
						34.0	51.0	14.9		59.5	40.5	
36′			A1298C:Glu430Ala	PCR-RFLP	241	*A/A*	*A/C*	*C/C*		*A*	*C*	
						65.1	31.1	3.7		80.7	19.3	
37	*Monoamine oxidase A* (*MAO-A*)	Xp11	30-bp VNTR	PCR	116^M^	*2*	*3*	*4*		*2*	*3*	
						0.9	60.3	38.8		1.1	59.7	
					123^F^	*3/3*	*3/other*	*others*		*4*	*5*	
						37.4	43.9	18.7		39.0	0.3	
38′	*NAD(P)H:quinone oxidoreductase* (*NQO1*)	Unknown	Pro187Ser	PCR-CTPP	241	*Pro/Pro*	*Pro/Ser*	*Ser/Ser*		*Pro*	*Ser*	
		(M81600)				35.7	44.4	19.9		57.9	42.1	
39	*oh8Gua glycosylase* (*OGG1*)	3p26.2	Ser326Cys	PCR-CTPP	240	*Ser/Ser*	*Ser/Cys*	*Cys/Cys*		*Ser*	*Cys*	
		(AC022382)				28.3	49.2	22.5		52.9	47.1	
40′	*p53*	17p13	Arg72Pro	PCR-CTPP	239	*Arg/Arg*	*Arg/Pro*	*Pro/Pro*		*Arg*	*Pro*	
		(X60016)				37.7	44.4	18.0		59.8	40.2	
41′	*p73*	1p36	GC/AT at exon 2	PCR-CTPP	235	*AT/AT*	*AT/GC*	*GC/GC*		*AT*	*GC*	
		(AL136528)				54.7	40.9	4.3		75.5	24.5	
42′	*Secretor* (*Se*, *FUT2*)	19q13.3	Se/se(sej, se5)	PCR-RFLP	239	*Se/Se*	*Se/se*	*se/se*		*Se*	*se*	
						25.5	53.1	21.3		52.1	47.9	
43	*Steroid 5α reductase type II* (*SRD5A2*)	Unknown	Ala49Thr	PCR-CTPP	240	*Ala/Ala*	*Ala/Th*r	*Thr/Thr*		*Ala*	*Thr*	
		(L03843)				100.0	0.0	0.0		100.0	0.0	
44			Val89Leu	PCR-RFLP	237	*Val/Val*	*Val/Leu*	*Leu/Leu*		*Val*	*Leu*	
						28.7	44.3	27.0	*	50.8	49.2	
45			2-bp VNTR	PCR	240	*0/0*	*0/9*	*9/9*		*0*	*9*	
						79.2	18.3	2.5	*	88.3	11.7	
46	*Transforming growth factor B1* (*TGF-B1*)	19q13.1	Leu10Pro	PCR-CTPP	115^F^	*Leu/Leu*	*Leu/Pro*	*Pro/Pro*		*Leu*	*Pro*	
		(X05839)				22.6	49.6	27.8		47.4	52.6	
47	*Tumor necrosis factor A* (*TNF-A*)	6p21.3	G-308A	PCR-CTPP	240	*G/G*	*G/A*	*A/A*		*G*	*A*	
		(X02910)				97.5	2.5	0.0		98.8	1.2	
48	*Tumor necrosis factor B* (*TNF-B*)	6p21.3	A252G	PCR-CTPP	241	*A/A*	*A/G*	*G/G*		*A*	*G*	
		(M55913)				36.5	48.1	15.4		60.6	39.4	
49	*XPD* (*ERCC2*)	19q13	Lys751Gln	PCR-RFLP	240	*Lys/Lys*	*Lys/Gln*	*Gln/Gln*		*Lys*	*Gln*	
						90.4	8.8	0.8	*	94.8	5.2	
50	*X-ray repair cross-complementing group 1* (*XRCC1*)	19q13.2	Arg399Gln	PCR-CTPP	241	*Arg/Arg*	*Arg/Gln*	*Gln/Gln*		*Arg*	*Gln*	
		(L34079)				47.7	44.8	7.5		70.1	29.9	

Hardy-Weinberg equilibrium test: * 0.05<p < 0.1, F: females, and M: males.

′ of No. shows the polymorphisms whose genotype frequency was reported in the previous papers.

DNA was extracted from buffy coat fractions by Qiagen QIAamp DNA Blood Mini Kit (QIAGEN Inc., Valencia, CA). Genotyping was conducted by PCR (polymerase chain reaction), PCR-RFLP (restriction fragment length polymorphism), or PCR-CTPP (PCR with confronting two-pair primers)^16^^)^. The primers and PCR conditions for PCR-RFLP were adopted from the published papers. Since the PCR-CTPP conditions were designed by the authors, the accession numbers in GenBank were attached in Table 1. All PCR products were visualized on a 2-4% agarose gel with ethidium bromide staining.

The Hardy-Weinberg equilibrium was tested by ‘genhwi’ of STATA version 7 (STATA Corp., College Station, TX). A chi-squared test was used for the association of genotype frequencies between two polymorphisms. The expected frequency for individuals with genotypes X_i_ and Y_j_ was calculated by X_i_×Y_j_×N, where X_i_ and Y_j_ are genotype frequency of i-th genotype of X polymorphism and j-th genotype of Y polymorphism, respectively, and N is the total number of participants genotyped for both X and Y polymorphisms.

## RESULTS

Table 1 shows the genotype/allele frequencies for the 50 polymorphisms; in total, 10,549 times of genotyping. Seven out of 50 polymorphisms were reported to be polymorphic in GenBank or studies for Caucasians, but actually not for the present Japanese subjects. The reported heterozygosity (1- sum(X_i_^2^), where X_i_ is the allele frequency of i-th allele) was 0.011 for *COX2* C-62G, 0.095 for *COX2* Glu488Gly, and 0.052 for *COX2* Val511Ala. Allele frequency was reported 0.13 for *T* allele of *IL-8* C74T, and no information for *IL-6* Ins/Del 17C (AF039337 for the insertion-type, and AF048692 for the deletion-type). *IL-1A* 46-bp VNTR was reported in a paper in the United Kingdom, which described that all subjects were with 5 repeats or over^17^^)^. Concerning *SRD5A2* Ala49Thr, whose *Thr* allele was not observed for Japanese so far^18^^)^, all the subjects had the *Ala/Ala* genotype. The exact binomial 95% confidence interval for 0 out of 100 chromosomes is 0-0.036, and that for 0 out of 200 chromosomes is 0-0.018.

A test of Hardy-Weinberg equilibrium showed marginally significant departure (0.05<p<0.1) for 7 polymorphisms, while the others were in Hard-Weinberg equlibrium.

To our knowledge, the genotype frequencies among Japanese were firstly reported in this paper for 15 polymorphisms; *CD36* A52C, *CXCR2* C785T, *CCND1* G870A, *IGF1* C/T at intron 2 and G2502T, *IL-1A* 46-bp VNTR, *IL-1R1* C-116T, *IL-6* Ins/Del 17C, *IL-8* T-278A and C74T, *IL-10* T-819C, *LEP* A-2548G, *SRD5A2* 2-bp VNTR, *XPD* Lys751Gln, and *XRCC1* Arg399Gln.

*IL-1* genes (*IL-1A*, *IL-1B*, and *IL-1RN*) make a cluster on chromosome 2q14. Tables 2 and 3 show the cross tabulations of *IL-1B* C-31T genotype with *IL-1A* C-889T and *IL-1RN* 86-bp VNTR, respectively. A statistically significant association was not observed for them. The most common genotype combination was *C/T* of *IL-1B* C-31T and *C/C* of *IL-1A* C-889T (45.2%), and *C/T* of *IL-1B* C-31T and *4/4* of *IL-1RN* 86-bp VNTR (51.0%). A tight linkage between *IL-1B* C-31T and C-511T was reported in our previous paper^8^^)^. Among the combinations of the four *IL-1* polymorphisms, individuals with *C/C* of *IL-1A* C-889T, *C/T* of *IL-1B* C-511T, *C/T* of *IL-1B* C-31T, and *4/4* of *IL-1RN* 86-bp VNTR were the most prevalent (40.1%), followed by those with *C/C* of *IL-1A* C-889T, *C/C* of *IL-1B* C-511T, *T/T* of *IL-1B* C-31T, and *4/4* of *IL-1RN* 86-bp VNTR (20.5%).

**Table 2.** Combined genotype frequency of *IL-1B* C-31T and *IL-1A* C-889T on chromosome 2q14.

*IL-1B*	*IL-1A*	Observed		Expected
			
C-31T	C-889T	N=241	%	N=241
*C/C*	*C/C*	35	14.5	35.0
*C/C*	*C/T*	6	2.4	6.7
*C/C*	*T/T*	1	0.4	0.1
*C/T*	*C/C*	109	45.2	110.9
*C/T*	*C/T*	24	9.9	21.5
*C/T*	*T/T*	0	0.0	0.5
*T/T*	*C/C*	57	23.6	55.0
*T/T*	*C/T*	9	3.7	10.6
*T/T*	*T/T*	0	0.0	0.2

* *χ*^2^=5.48, d.f.=4, p=0.241

**Table 3.** Combined genotype frequency of *IL-1B* C-31T and *IL-1RN* 86-bp VNTR on chromosome 2q14.

*IL-1B*	*IL-1RN*	Observed		Expected
			
C-31T	VNTR	N=241	%	N=241
*C/C*	*4/4*	35	14.5	37.8
*C/C*	*4/other*	6	2.4	3.8
*C/C*	*others*	1	0.4	0.3
*C/T*	*4/4*	123	51.0	119.7
*C/T*	*4/other*	9	3.7	12.1
*C/T*	*others*	1	0.4	1.1
*T/T*	*4/4*	59	24.4	59.4
*T/T*	*4/other*	7	2.9	6.0
*T/T*	*others*	0	0.0	0.5

* *χ*^2^=4.27, d.f.=4, p=0.371

Tables 4 to 7 show the cross tabulations for *IL-1B* C-31T and *IL-1R1* C-116T on chromosome 2q, for Val89Leu and 2-bp VNTR of *SRD5A2*, for *TNF-A* G-308A and *TNF-B* A252G on chromosome 6p21.3, and for *XPD* Lys751Gln and *XRCC1* Arg399Gln on chromosome 19q13, respectively. A statistically significant association was found for the combinations between *SRD5A2* Val89Leu and 2-bp VNTR, and between *TNF-A* G-308A and *TNF-B* A252G. In *SRD5A2*, *89Leu* and *9 repeats* haplotype was not observed, and in *TNFs A* allele of *TNF-A* and *A* allele of *TNF-B* was not.

**Table 4.** Combined genotype frequency of *IL-1B* T-31C and *IL-1R1* C-116T on chromosome 2q.

*IL-1B*	*IL-1R1*	Observed		Expected
			
C-31T	C-116T	N=239	%	N=239
*C/C*	*C/C*	17	7.1	16.3
*C/C*	*C/T*	22	9.2	20.0
*C/C*	*T/T*	3	1.2	5.6
*C/T*	*C/C*	59	24.6	51.7
*C/T*	*C/T*	56	23.4	63.4
*C/T*	*T/T*	18	7.5	17.8
*T/T*	*C/C*	17	7.1	24.9
*T/T*	*C/T*	36	15.0	30.5
*T/T*	*T/T*	11	4.6	8.5

* *χ*^2^=7.51, d.f.=4, p=0.111

**Table 5.** Combined genotype frequency of *SRD5A2* Val89Leu and 2-bp VNTR.

*SRD5A2*		Observed		Expected
		
Val89Leu	2-bp VNTR	N=236	%	N=236
*Val/Val*	*0/0*	40	16.9	53.8
*Val/Val*	*0/9*	22	9.3	12.3
*Val/Val*	*9/9*	6	2.5	1.7
*Val/Leu*	*0/0*	83	35.1	82.4
*Val/Leu*	*0/9*	21	8.8	18.9
*Val/Leu*	*9/9*	0	0.0	2.6
*Leu/Leu*	*0/0*	64	27.1	50.7
*Leu/Leu*	*0/9*	0	0.0	11.6
*Leu/Leu*	*9/9*	0	0.0	1.6

* *χ*^2^=41.2, d.f.=4, p<0.001

**Table 6.** Combined genotype frequency of *TNF-A* G-308A and *TNF-B* A252G on chromosome 6p21.3.

*TNF-A*	*TNF-B*	Observed		Expected
			
G-308A	A252G	N=240	%	N=240
*G/G*	*A/A*	88	36.6	85.8
*G/G*	*A/G*	112	46.6	112.1
*G/G*	*G/G*	34	14.1	36.0
*G/A*	*A/A*	0	0.0	2.2
*G/A*	*A/G*	3	1.2	2.8
*G/A*	*G/G*	3	1.2	0.9

* *χ*^2^=10.0, d.f.=2, p=0.007

**Table 7.** Combined genotype frequency of *XRCC1* Arg399Gln and *XPD* Lys751Gln on chromosome 19q13.

*XRCC1*	*XPD*	Observed		Expected
			
Arg399Gln	Lys751Gln	N=240	%	N=240
*Arg/Arg*	*Lys/Lys*	100	41.6	103.9
*Arg/Arg*	*Lys/Gln*	14	5.8	10.0
*Arg/Arg*	*Gln/Gln*	1	0.4	0.9
*Arg/Gln*	*Lys/Lys*	101	42.0	96.7
*Arg/Gln*	*Lys/Gln*	5	2.0	9.3
*Arg/Gln*	*Gln/Gln*	1	0.4	0.8
*Gln/Gln*	*Lys/Lys*	16	6.6	16.2
*Gln/Gln*	*Lys/Gln*	2	0.8	1.5
*Gln/Gln*	*Gln/Gln*	0	0.0	0.1

* *χ*^2^=4.20, d.f.=4, p=0.380

## DISCUSSION

Microarray technology was invented to apply massive polymorphism genotyping. However, there are no papers reporting the genotype/allele frequencies so far. There are neither archival reports of genotype frequencies for the same subjects, while papers describing the genotype/allele frequencies for different study subjects have been published for many polymorphisms^19^^-^^21^^)^. This was the first paper reporting as many as 50 polymorphisms for the same Japanese subjects. The purpose of this paper was simply to provide basic information for the frequencies.

To date, the genotype/allele frequencies among Japanese controls other than the present subjects have been reported for *ALDH2* Glu487Lys (the largest study was with n=264)^22^^)^, *BAR2* Gln27Glu (n=149)^23^^)^, *BAR3* Trp64Arg (n=553)^24^^)^, *COMT* Val158Met (n=150)^25^^)^, *CD36* Pro90Ser (n=100)^26^^)^, *CYP2A6* Wt/Del/Conv (n=201)^27^^)^, *CYP17* T-34C (n=195)^28^^)^, *CYP19* Trp39Arg (n=199)^29^^)^, *IL-1B* C-511T (n=112)^30^^)^, *IL-1RN* 86-bp VNTR (n=65)^31^^)^, *Le* Le/le1/le2/le3 (n=400)^32^^)^, *L-myc* L/S (n=107)^33^^)^, *MTHFR* C677T (n=778)^34^^)^, *MAO-A* 30-bp VNTR (n=254)^35^^)^, NQO1 Pro187Ser (n=150)^36^^)^, *OGG1* Ser326Cys (n=197)^37^^)^, *Se* Se/sej/se5 (n=400)^32^^)^, *p53* Arg72Pro (n=110)^38^^)^, *SRD5A2* Ala49Thr (n=181) and Val89Leu (n=203)^18^^)^, *TGF-B* Leu10Pro (n=591)^39^^)^, *TNF-A* G-308A (n=575)^1^^)^, and *TNF-B* A252G (n=165)^40^^)^. Concerning the allele linkages among other ethnic groups, there have been several studies reported. For the IL-1s, the tight linkage between *IL-1B* C-31T and C-511T was similarly reported for Caucasians^41^^)^. The haplotype of *TNF-A* G-308A (described as *TNF* -308) and *TNF-B* A252G (*LTα*NcoI) was also reported for Australian general population^42^^)^. Information on the linkage is useful in epidemiologic studies 1) for the interpretation of observed associations, 2) to avoid dual genotyping, and 3) to understand superficially inconsistent findings among different ethnic groups.

Since the present subjects were outpatients, they might not reflect general inhabitants residing in and around Aichi prefecture. Ninety-seven out of 241 participants stated that they were under medication. Forty-six of the 97 participants had digestive ulcer or gastritis, so the potential influence should be discussed. Since data on medication in a randomly sampled general population with a similar sex-age distribution were not available, we were not able to comment the comparability between the present subjects and general population. However, even if the genotype distribution of some polymorphisms among the present subjects under medication were different from the rest of the subjects, the influence seemed to be limited. The reasons were 1) if exists, the odds ratio of polymorphism for the medicated subjects should be small due to two reasons; heterogeneous diseases were included, and the strong association between the listed common diseases and polymorphisms seemed unlikely, 2) the effect was diluted in the whole 241 subjects, and 3) the random error might be larger than the systematic bias due to the moderate sample size. The 95% confidence interval of genotype frequency for 240 subjects is, for examples, 6.5-14.5% for 10%, 24.3-36.2% for 30%, and 43.5-56.5% for 50%, while a 10% higher genotype among 46 (digestive ulcer + gastritis) out of 240 is diluted into a 1.9% of elevation of the genotype frequency among the whole 240 subjects, though this size of bias should be in mind for the interpretation of the present genotype frequencies.

All the polymorphisms were genotyped between November 1999 and September 2001 in Aichi Cancer Center Research Institute. The method was PCR for 4 VNTR polymorphisms, PCR-RFLP for 15 polymorphisms, and PCR-CTPP for 30 SNPs and one Ins/Del polymorphism. In our Division, about 1,000 samples per week have been genotyped by four technicians in the past year without massive genotyping method such as microarray. By using PCR-CTPP, the genotyping speed has been accelerated; both the costs and time became half of the PCR-RFLP. At this point of time, PCR-CTPP seems to be the most efficient method for ordinal genotyping laboratories^43^^)^, although the PCR condition should be carefully determined^44^^)^.

In association studies on genotype frequencies, a case-control design is usually adopted. The controls should be sampled from the individuals without the disease under study in the same population from which the cases are sampled. In the confirmative studies, subjects are to be sampled according to a study protocol prepared before the start. Meanwhile, to make hypotheses, case studies and comparisons with available comparable controls are conducted. The present genotype frequencies can be used for such hypothesis making. Since distributions of potential risk factors have been regarded as important sources of information in descriptive epidemiology, the genotype/allele frequencies listed in this paper can also play an important role in future epidemiologic studies.
